# Decoding Motor States from Phase–Amplitude Coupling Measured by OPM-MEG

**DOI:** 10.3390/bios16060338

**Published:** 2026-06-15

**Authors:** Yong Li, Hao Lu, Min Xiang, Jianzhi Yang, Binyi Su, Fuzhi Cao

**Affiliations:** 1 Key Laboratory of Ultra-Weak Magnetic Field Measurement Technology, Ministry of Education, School of Instrumentation and Optoelectronic Engineering, Beihang University, Beijing 100191, China; by2017335@buaa.edu.cn (Y.L.);; 2 Zhejiang Provincial Key Laboratory of Ultra-Weak Magnetic-Field Space and Applied Technology, Hangzhou 310051, China; 3 Zhejiang Key Laboratory of Zero Magnetic Medicine, Hangzhou 310006, China; 4 State Key Laboratory of Traditional Chinese Medicine Syndrome, National Institute of Extremely-Weak Magnetic Field Infrastructure, Hangzhou 310028, China; 5 Hefei National Laboratory, Hefei 230088, China; 6 School of Artificial Intelligence and Data Science, Hebei University of Technology, Tianjin 300130, China

**Keywords:** optically pumped magnetometer, OPM-MEG, phase–amplitude coupling, motor state decoding, motor control

## Abstract

Optically Pumped Magnetometers (OPMs) have emerged as a promising technology for developing flexible, wearable magnetoencephalography (OPM-MEG) systems, offering high spatiotemporal resolution without the need for cryogenic cooling. However, their application to phase–amplitude coupling (PAC)-based neural decoding remains largely unexplored. Investigating their decoding performance is essential for evaluating the capability of OPM-MEG in characterizing complex neural dynamics and discriminating motor states. In this study, OPM-MEG was utilized to record brain activity during rest, motor imagery, and motor execution tasks. A two-stage temporal optimization strategy combining time-resolved PAC localization and the Kullback–Leibler modulation index (KL-MI) was employed to extract robust PAC features from low-frequency phase and high-frequency amplitude coupling. α–γ and θ–γ PAC features were subsequently fed into a multiclass linear discriminant analysis (LDA) classifier for motor state decoding, and compared against baseline band-power feature decoding performance. Experimental results demonstrate that PAC features derived from OPM-MEG significantly outperform the corresponding baseline band-power features in decoding performance. Notably, α–γ PAC features effectively discriminate among different motor states, achieving a balanced accuracy of 85.91% in 10-fold cross-validation. This performance significantly exceeds the 50% one-vs-rest chance level and outperforms θ–γ PAC features. These findings provide initial evidence for the feasibility of OPM-MEG in PAC-based motor state decoding and a preliminary case study for characterizing motor-related neural dynamics in a wearable MEG system.

## 1. Introduction

In recent years, driven by rapid advances in quantum sensing technology, optically pumped magnetometers (OPMs) based on the spin-exchange relaxation-free (SERF) principle have progressively moved toward practical commercialization [1]. Owing to their high spatiotemporal resolution in measuring extremely weak neuromagnetic signals, OPMs have been employed to construct high-precision magnetoencephalography systems (OPM-MEG) [2]. Compared with conventional superconducting quantum interference device (SQUID)-based MEG systems, OPM-MEG does not require cryogenic liquid helium cooling and offers wearable, flexible sensor configurations. These advantages improve signal quality and enhance experimental flexibility, enabling MEG recordings across a broader range of populations and dynamic task scenarios [3]. To date, OPM-MEG has been applied in various research domains, including sensory processing [4], functional connectivity analysis [5], brain–computer interfaces [6], and disease monitoring [7].

Due to its ultra-high sensitivity and superior spatiotemporal resolution, OPM-MEG has been demonstrated to capture one of the key neural dynamical markers of brain activity—phase–amplitude coupling (PAC) [8]. While that prior work established the technical feasibility of measuring PAC with OPM-MEG, it did not assess whether these PAC features carry sufficient task-relevant information to discriminate motor states. PAC refers to the mechanism by which the phase of low-frequency oscillations modulates the amplitude of high-frequency oscillations, and is considered an important neural coding strategy for cross-timescale information integration and inter-regional communication [9]. Previous studies have shown that PAC plays a critical role in motor-related neural encoding [10,11,12]. In particular, during motor imagery and motor execution, the modulation of high-frequency activity (e.g., γ band) by low-frequency rhythms (e.g., δ, θ, and α bands) is closely associated with cortical motor function [13,14,15].

However, the discriminative capability of OPM-MEG-derived PAC features across different motor paradigms, such as motor imagery and motor execution, remains unclear. Moreover, the decoding performance of specific low- and high-frequency band combinations requires systematic investigation. Therefore, the present study applies PAC features derived from OPM-MEG to motor state decoding for the first time, systematically evaluating whether cross-frequency coupling features can discriminate among rest, motor imagery, and motor execution states. Utilizing PAC features derived from OPM-MEG for motor-state decoding not only contributes to a deeper understanding of neural information integration mechanisms during motor control but also provides a normative decoding reference for the non-invasive assessment of abnormal cross-frequency coupling patterns associated with movement disorders. This approach holds potential clinical value for future investigations of motor-related neurological diseases, such as Parkinson’s disease [16].

To evaluate the motor-state decoding performance of PAC features measured by OPM-MEG, we first designed motor imagery and motor execution paradigms and recorded brain signals under both conditions using OPM-MEG. We then characterized the fundamental PAC patterns within motor-related cortical regions to establish a foundation for subsequent decoding analysis. Finally, a multiclass linear discriminant analysis (LDA) model was employed for PAC-based motor-state decoding, demonstrating that α–γ PAC features measured by OPM-MEG enable high-performance motor-state classification.

## 2. Materials and Methods

### 2.1. Participants and Experimental Paradigm

Ten healthy volunteers (eight males and two females, aged 24 to 30 years, mean ± SD: 27.8 ± 2.07 years) participated in this study. All participants were right-handed native Chinese speakers and had no history of congenital developmental disorders, hearing impairment, motor dysfunction, neurological diseases, or psychiatric illnesses. Written informed consent was obtained from each participant before their involvement in the study. The study protocol received approval from the Biomedical Ethics Committee of Beihang University and was conducted in accordance with the ethical principles outlined in the Declaration of Helsinki.

A priori power analysis was conducted using G*Power 3.1.9.7 to estimate the required sample size based on Cohen’s statistical power theory. Informed by the average effect sizes reported in comparable neural decoding studies, we targeted a Cohen’s f of 0.5. For a within-subject repeated-measures ANOVA across three conditions, the calculation indicated that 10 participants would provide a statistical power exceeding 0.80 (calculated power = 0.835), assuming a significance level of (α = 0.05) and a correlation among repeated measures of (ρ = 0.5). Our experimental results subsequently validated this estimation, yielding an observed partial eta-squared (η^2^_p_) of 0.245 (corresponding to f = 0.57). This resulted in a post hoc power of 0.852, suggesting that the current sample size was appropriate for the exploratory objectives of this study.

**Figure 1:**
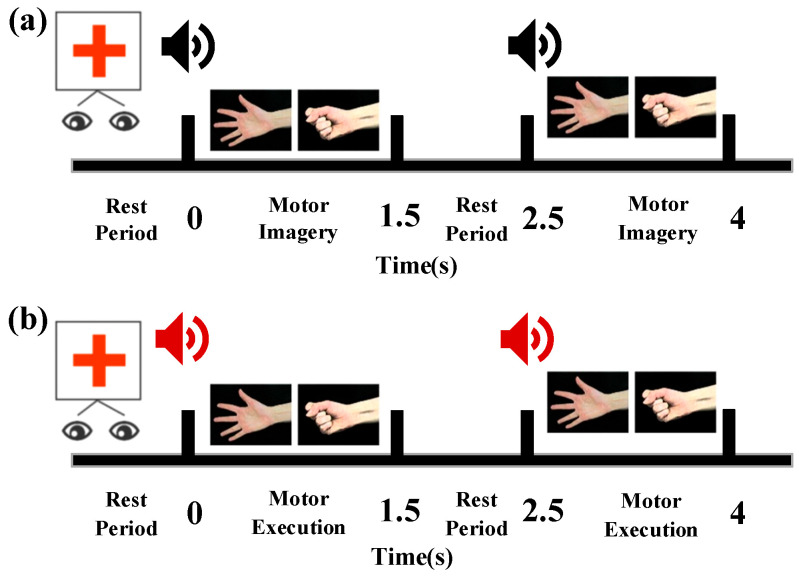
Experimental design of the motor state tasks: (**a**) motor imagery task; (**b**) motor execution task.

To investigate PAC during different motor states, a motor imagery and motor execution paradigm was designed based on previous literature [14]. A schematic illustration of the experimental paradigm is shown in Figure 1. The experiment consisted of two modules: (a) a motor imagery task and (b) a motor execution task. For each participant, both tasks were performed within a single experimental session on the same day to ensure inter-task stationarity. During all experimental sessions, participants kept their eyes open and were instructed to fixate on a red cross to minimize eye-movement artifacts and maintain attention. In the motor imagery condition, participants began imagining a right-hand grasping movement upon hearing an auditory cue (1000 Hz, 64 dB), which was delivered at a comfortable intensity level; the imagery period lasted 1.5 s. In the motor execution condition, participants performed an actual right-hand grasping movement upon hearing the same auditory cue and were required to complete the movement within 1.5 s. Prior to the formal experiment, practice trials were conducted to ensure that participants understood and could perform the tasks correctly. Each task comprised two blocks of 100 trials each (200 trials in total), with a 5-min rest interval between blocks. To eliminate potential order effects and fatigue-related bias, the sequence of the two task modules was counterbalanced across participants; specifically, five participants initiated the experiment with the motor imagery task, while the remaining five started with the motor execution task.

### 2.2. Data Acquisition and Preprocessing

**Figure 2:**
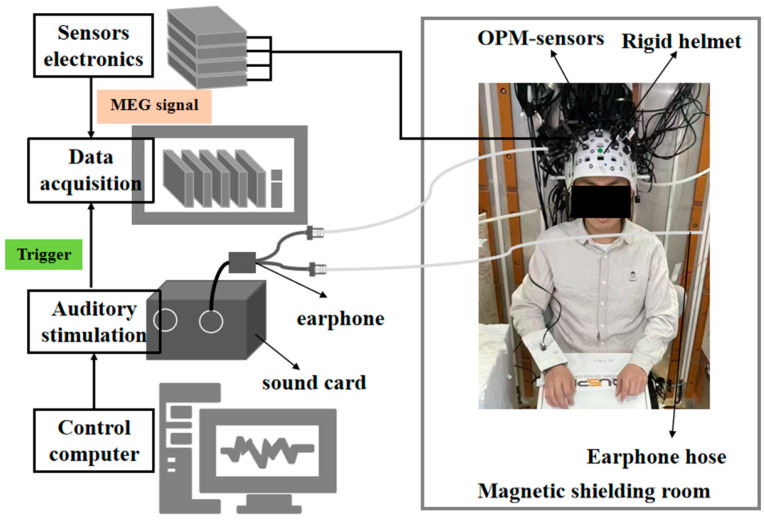
OPM-MEG system.

**Figure 3:**
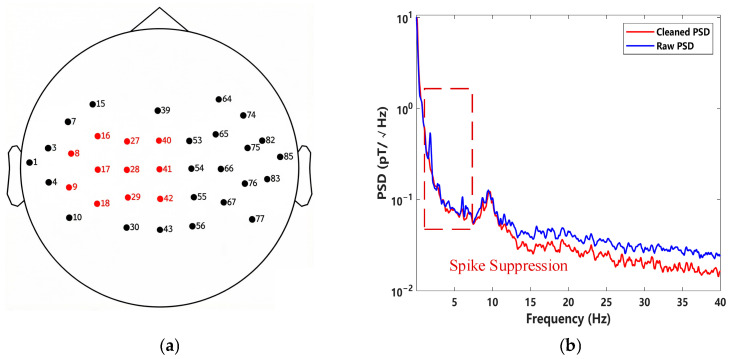
Average power spectrum of all marker sensors for a representative subject: (**a**) Sensor layout and sensors covering the region of interest (Red); (**b**) the average power spectrum of the 11 sensors covering the region of interest.

The experimental data for the motor imagery and motor execution tasks were acquired using the OPM-MEG system shown in Figure 2. This system utilized 35 s-generation optically pumped magnetometers (Quspin Inc., Louisville, CO, USA) to measure radial neuromagnetic signals from cortical regions, including the bilateral temporal lobes, precentral gyrus, postcentral gyrus, and superior frontal gyrus. To ensure a constant sensor-to-scalp distance and minimize movement-related artifacts, all 35 OPM sensors (Figure 3a) were securely housed in a custom-designed, rigid 3D-printed nylon helmet (Figure 2). This configuration ensured that the sensors remained stationary relative to the participant’s head during all tasks. Furthermore, participants were trained and instructed to maintain a steady head position and perform the motor execution task (e.g., finger movements) with minimal involvement of proximal muscle groups. MEG signals were recorded using a PXI-based computer (PXIC-7318C, ART Technology Inc., Beijing, China) at a sampling rate of 1000 Hz, with a 16-bit analog-to-digital converter. The experiment was conducted at the center of a magnetically shielded room (MSR), where the residual magnetic field was below 10 nT and the magnetic field gradient was less than 0.1 nT/cm. The extremely low residual magnetic field and gradient further mitigated the induction of artifacts caused by residual micro-movements of the head. During the experiment, auditory pitch sequences were generated and controlled using Psychology Toolbox software 3.0.18 integrated into the stimulus system. The sounds were delivered via headphones and transmitted to the MSR via plastic tubing to minimize interference from headphone currents in the MEG recordings.

In this study, uniform field correction was first applied to reduce interference from distant noise sources in the frequency spectrum. To suppress unknown narrowband noise, particularly near 25 Hz and 60 Hz, spectral interpolation techniques were used to restore the affected frequency components. All data were then band-pass filtered between 2 and 100 Hz using a fourth-order zero-phase Butterworth filter (applied bidirectionally to prevent phase distortion), implemented using the SciPy library (version 1.11.4) in Python 3.9.13. Additionally, a 50 Hz notch filter was applied to further remove power-line noise for both task conditions. Subsequently, eye-blink and cardiac artifacts were removed using an automated physiological artifact correction model as described in [17]. Following this, the continuous data were segmented into epochs, each spanning from 1.0 s before to 1.5 s after the onset of the auditory cue, resulting in epochs of 2.5 s in total. To reduce transient artifacts potentially caused by subtle head movements or sensor drifts, a sliding window of 0.5 s with 50% overlap was then employed to calculate the standard deviation (STD) for each extracted epoch. If the STD within any window during stimulus presentation exceeded three times the average STD of that window across all epochs, the corresponding epoch was rejected. This procedure effectively eliminated the influence of artifact-contaminated trials associated with head movement. Specifically, following artifact suppression, we visualized the average power spectrum of the 11 sensors covering the region of interest, as illustrated in Figure 3; it is evident that the artifact spikes in the low-frequency bands were effectively suppressed. Finally, after this complete preprocessing and artifact rejection pipeline, 160 trials remained for the final decoding analysis.

### 2.3. Feature Extraction and Decoding Analysis

#### 2.3.1. Registration and Source Reconstruction

To obtain neural activity with high spatial fidelity and physiological relevance, the OPM-MEG recordings were projected from the sensor level into the source space. First, the relative positions and orientations of the OPM sensors were determined by co-registering 3D digitized sensor locations with the scalp surface extracted from individual T1-weighted MRI data. Following the methodology described in [18], a transformation matrix was calculated to map the sensor coordinates into the MRI space, providing a unified spatial framework for forward modeling.

Cortical reconstruction was then performed using FreeSurfer 7.4.1, where the scalp, skull, and brain tissues were segmented via a watershed algorithm to construct a three-layer Boundary Element Model (BEM) [19]. The source space was defined within the MRI coordinate system, and the forward solution was computed by combining the transformation matrix with the BEM [20]. The noise covariance matrix was estimated from baseline data collected between −1.0 s and −0.5 s. Finally, the source distribution was determined using depth-weighted minimum norm estimation (dMNE) [21], with the depth weighting parameter set to 0.8 [22]. During reconstruction, results from all subjects were morphed onto the “fsaverage” brain template [23] to ensure standardized alignment across the group.

**Figure 4:**
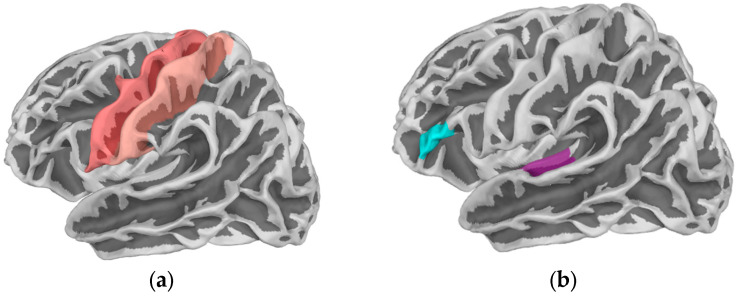
Brain region Selection. (**a**) Brain regions of interest. (**b**) brain region of reference.

To extract PAC features from the regions of interest (ROIs), we defined the anatomical boundaries of the target motor cortex based on the methodology established in [24], as illustrated in Figure 4a. This ROI comprises Brodmann Area 4 (BA4) and Brodmann Area 6 (BA6), which together encapsulate the core components of the motor cortex. Specifically, BA4 represents the primary motor cortex, which is directly responsible for executing voluntary movements, particularly fine motor control of the fingers; BA6 constitutes the premotor and supplementary motor areas, which are extensively involved in motor planning, sensory-guided actions, and higher-order motor cognition. Additionally, to analyze whether the PAC features were induced by background field changes caused by head movements, the inferior frontal gyrus (IFG) and auditory regions were selected as reference areas for comparative analysis, as illustrated in Figure 4b. For these designated regions, the representative neural time series signals were extracted by calculating the first principal component [8] via principal component analysis (PCA) of all reconstructed dipoles within their anatomical boundaries for subsequent decoding analysis. This systematic approach ensures that the subsequent analysis is grounded in biologically meaningful cortical signals, thereby providing a robust foundation for motor state decoding.

#### 2.3.2. Decoding Feature Extraction

To capture the transient and non-stationary nature of PAC during motor tasks, we implemented a two-stage temporal optimization strategy combining time-resolved localization and robust feature extraction. The procedure began by filtering the preprocessed MEG data into the frequency bands of interest: δ (2–4 Hz), θ (4–8 Hz), α (8–12 Hz), and γ (30–100 Hz).

In the first stage (Temporal Localization), the time-resolved PAC (tPAC) algorithm [25] was implemented to capture the transient dynamics of PAC and avoid the loss of temporal information inherent in averaging over a non-stationary window. By characterizing the dynamic evolution of coupling strength, we identified the peak-coupling interval (e.g., 100–600 ms post-cue) from the resulting time-varying PAC. This localization ensures that the analysis is constrained to the most task-relevant period, effectively pruning non-informative segments from the full time window.

In the second stage (Feature Refinement), the Kullback–Leibler modulation index (KL-MI) method [26] was employed within this optimized temporal window to quantify the robust coupling strength. Specifically, the Hilbert transform was applied to extract the instantaneous phase of the low-frequency bands (δ, θ, and α) and the amplitude envelope of the γ band. Based on these analytic signals, the phase values within the modulating frequency range $f_{p}$ (−180° to 180°) were divided into 18 bins of 20° each. For each phase bin, the normalized mean amplitude of the modulated frequency range $f_{A}$ was calculated:

(1) $$P(j)=\frac{\overset{-}{A_{f_{A}}}(j)}{\sum_{k=1}^{N}A_{f_{A}}(k)}$$

Here, $\overset{-}{A_{f_{A}}}(j)$ denotes the mean amplitude within the j-th phase bin, and N represents the total number of bins. The PAC value was then quantified using the modulation index method based on the Kullback–Leibler (KL) divergence, as defined in Equation (2):

(2) $$MI=\frac{D_{\mathrm{KL}}(P)}{\log(N)}$$

In this formulation, $D_{KL}(P)$ represents the KL divergence, which quantifies the deviation between the empirical phase–amplitude distribution and a uniform distribution. It is computed according to Equation (3):

(3) $$D_{\mathrm{KL}}(P)=\log(N)-H(P)$$

Here, the uniform distribution is represented by log(N), while the empirical amplitude distribution across phase bins is quantified using the Shannon entropy H(P), defined as:

(4) $$H(P)=-\sum_{j=1}^{N}P(j)\log[P(j)]$$

The rationale for calculating KL-MI within the tPAC-optimized window, rather than using the raw tPAC time-series for decoding, is that KL-MI captures the cumulative phase-amplitude distribution to represent a stable neural state. Unlike raw instantaneous values, which are highly susceptible to point-wise noise and trial-to-trial variability, KL-MI quantifies the robust coupling profile of the motor task. By summarizing the data within the optimized window, this approach filters out high-frequency temporal noise and substantially reduces the feature dimensionality. Consequently, it avoids the risk of model overfitting that typically arises from high-dimensional, noisy time-series data, thereby enhancing classification stability.

After quantifying the PAC values using the KL-MI method, surrogate data were generated under the null hypothesis of random coupling by randomly shuffling the phase and amplitude trial data [27]. For each trial, 1000 surrogate datasets were created using this randomization procedure, and the corresponding surrogate PAC values ($MI_{\mathrm{Surrogate}}$) were calculated. Finally, the MI values were transformed into z-scores according to Equation (5) to reduce scale-related variability and minimize magnitude bias during classification.

(5) $$MI_{z}=\frac{MI-\mathrm{average}\left(MI_{\mathrm{Surrogate}}\right)}{\mathrm{std}\left(MI_{\mathrm{Surrogate}}\right)}$$

The group-level PAC maps were visualized by averaging the responses across all trials.

To facilitate a direct comparison between PAC and baseline power features, this study utilized the Hilbert transform for time-frequency decomposition to characterize the power profiles. Specifically, instantaneous power was derived by squaring the amplitude envelope of the analytical signal [28]. During this power calculation, the 4–100 Hz spectrum was discretized into non-overlapping 2 Hz sub-bands to compute the instantaneous power values.

For the decoding analysis, based on the statistical significance analysis results, the rest period feature extraction window for all features was defined from −500 to 0 ms relative to cue onset. The normalized PAC values were computed within the designated feature extraction windows for each experimental condition. During the PAC feature extraction stage, the feature extraction windows for motor imagery and motor execution tasks were set from 100 to 600 ms for α–γ features and from 600 to 1100 ms for θ–γ features, both corresponding to their respective periods of significant difference across the distinct task states. Similarly, based on the analysis of significant differences between task states, the extraction window for band power features during motor imagery and motor execution tasks was defined as 300–1200 ms. Specifically, the mean power within the specified time window was used as the power feature value corresponding to that window. For further details regarding the statistical significance analysis, please refer to Section 3.1.

#### 2.3.3. Feature Decoding Method

In this study, the feature decoding problem is formulated as a three-class classification problem aimed at identifying different motor states. To ensure that the decoding performance was primarily driven by the intrinsic properties of the neural features rather than classifier-specific biases, we benchmarked three distinct algorithmic paradigms: Linear Discriminant Analysis (LDA) [29], Support Vector Machines (SVM), and Random Forests (RF). The evaluation details are summarized in Appendix A Table A1, which demonstrates a highly consistent performance trend across all three classifiers for each feature type. Consequently, LDA was selected for the final decoding analysis due to its high computational efficiency and rapid execution speed. Furthermore, to demonstrate the efficacy of PAC-based decoding, traditional power features were employed as a baseline for comparative analysis to highlight the distinct advantages of PAC in characterizing motor states.

The underlying principle of the LDA model is to characterize the distribution of samples by defining the within-class scatter matrix $S_{W}$ and the between-class scatter matrix $S_{B}$:

(6) $$S_{W}=\sum_{j=1}^{c}\sum_{x_{i}\in C_{j}}(x_{i}-\mu_{j}){\left(x_{i}-\mu_{j}\right)}^{Τ}$$

(7) $$S_{B}=\sum_{j=1}^{c}N_{j}(\mu_{j}-\mu_{c}){\left(\mu_{j}-\mu_{c}\right)}^{Τ}$$

Here, $x_{i}$ denotes the i-th data sample, $\mu_{j}$ represents the mean of samples in the j-th class, $C_{j}$ represents the samples of class j-th. $N_{j}$ is the total number of samples in the j-th class, $\mu_{c}$ represents the overall sample mean across all classes, and T indicates vector transposition. Subsequently, a linear projection matrix W is determined by maximizing the ratio of between-class scatter to within-class scatter, thereby obtaining a discriminative subspace for multiclass classification:

(8) $$\underset{W}{\max}\frac{\left|W^{Τ}S_{B}W\right|}{\left|W^{Τ}S_{W}W\right|}$$

The solution to this problem can be reformulated as a generalized eigenvalue decomposition problem, i.e.,

(9) $$S_{B}W=\lambda S_{W}W$$

The optimal discriminative projection matrix W is then constructed by selecting the eigenvectors corresponding to the largest c−1 eigenvalues. Subsequently, the PAC feature vector of each trial is projected onto the discriminative subspace:

(10) $$z_{i}=W^{Τ}x_{i}$$

Within this low-dimensional discriminative space, PAC features corresponding to different motor states achieve maximal separability in a statistical sense. Under the shared covariance assumption, classification is performed by computing linear discriminant functions for each class and assigning each sample to the class with the highest discriminant score, thereby enabling multiclass motor state decoding.

To comprehensively evaluate the decoding performance and generalizability of PAC features, two cross-validation schemes were employed in this study.

(1) Within-subject 10-fold cross-validation: To assess the decoding capacity of PAC features within individual subjects, a 10-fold cross-validation scheme [30] was employed. Specifically, the dataset was pseudo-randomly partitioned into 10 equally sized subsets: nine subsets were used to train the classifier, while the remaining subset served as the test set. This process was repeated 10 times, ensuring that each sample in the dataset was used exactly once for testing and at least once for training, but never for both simultaneously. This scheme evaluates whether task-relevant information is present in the PAC features at the individual level.

(2) Leave-one-subject-out (LOSO) cross-validation: To address potential subject-specific variance and assess cross-subject generalizability of the neural features, a LOSO scheme was performed. In each iteration, data from one participant were held out as the independent test set, while the multiclass LDA classifier was trained on data from all remaining participants. This process was repeated until each participant served as the test set exactly once. This scheme is designed to evaluate the generalizability of the observed PAC patterns across subjects.

Finally, to assess model performance under both validation schemes, several evaluation metrics were adopted, including balanced accuracy, accuracy (one-vs-rest), recall, precision, and specificity, as well as the macro-average scores calculated from the class-average scores of the aforementioned metrics. These metrics were used to comprehensively evaluate the effectiveness of the proposed feature-based motor state decoding, which was implemented via the OPM-MEG system.

(11) $$\mathrm{balanced}\;\mathrm{accuracy}=\frac{1}{2}\frac{TP}{TP+FN}+\frac{1}{2}\frac{TN}{TN+FP}$$

(12) $$\mathrm{accuracy}=\frac{TP+TN}{TP+TN+FP+FN}$$

(13) $$\mathrm{recall}=\frac{TP}{TP+FN}$$

(14) $$\mathrm{precision}=\frac{TP}{TP+FP}$$

(15) $$\mathrm{specificity}=\frac{TN}{TN+FP}$$

Here, TP, FN, TN and FP denote the numbers of true positives, false negatives, true negatives, and false positives, respectively.

#### 2.3.4. Statistical Analysis

To identify statistically significant feature patterns, we employed a cluster-based nonparametric permutation test [31]. At the group level, we first computed the cross-trial average of time–frequency features for each participant under each experimental condition. Within-condition effects were then evaluated using a cluster-based one-sample permutation test with 10,000 sign-flipping permutations, assessing whether feature values in each condition significantly deviated from zero. Between-condition differences were examined by applying a cluster-based one-sample permutation test (also with 10,000 sign-flipping permutations) to the within-participant difference scores. For both types of tests, adjacent data points in feature space exceeding the cluster-forming threshold (*p* < 0.10) were grouped into a single cluster. A cluster was considered statistically significant only if its cluster-level corrected *p*-value was below 0.05.

To rigorously compare decoding performance across the two PAC feature types (θ–γ and α–γ), Wilcoxon signed-rank tests were performed on all performance metrics (accuracy, precision, recall, and F1 score) under both cross-validation schemes. For all pairwise comparisons, *p*-values were adjusted using Bonferroni correction to control for multiple comparisons.

## 3. Results

### 3.1. Visualization of PAC Analysis Results Based on OPM-MEG

**Figure 5:**
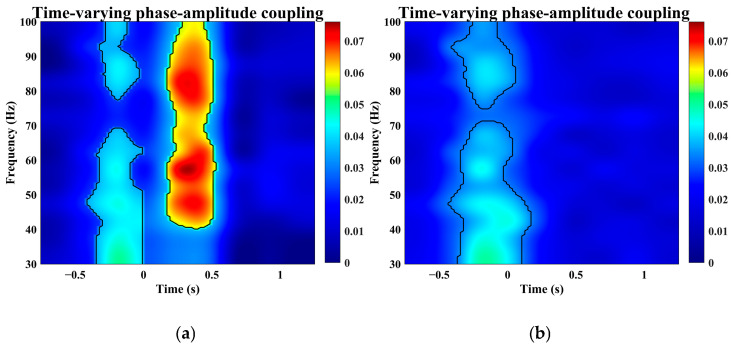
Grand average time-varying α–γ PAC across all subjects during motor imagery and motor execution tasks. (**a**) α–γ coupling during the motor imagery task. (**b**) α–γ coupling during the motor execution task.

Figure 5 illustrates the grand average time-varying α–γ PAC across all subjects during motor imagery and motor execution tasks. The horizontal axis represents time; given a sliding window of 0.5 s with a 50% overlap, the window centers range from −0.75 s to 1.25 s. The vertical axis corresponds to the selected high-frequency γ band (30–100 Hz), and the color scale indicates coupling strength. The black solid contours delineate the statistically significant time-frequency clusters derived from the nonparametric permutation tests (cluster corrected, *p* < 0.05).

In Figure 5a, which displays the α–γ coupling during the motor imagery task condition, two statistically significant clusters are observed during the rest period from −0.5 s to 0 s (cluster corrected, *p* < 0.05), spanning the high-frequency ranges of 35–68 Hz and 78–100 Hz, respectively. Crucially, during the motor imagery state from 0.1 s to 0.6 s, a highly significant cluster is observed (cluster corrected, *p* < 0.01), which extensively covers the high-frequency range from 40 Hz to 100 Hz. By contrast, Figure 5b presents the alpha –gamma coupling under the motor execution task condition. Similarly, two statistically significant clusters are observed during the rest period (cluster corrected, *p* < 0.05). However, distinct from the motor imagery task, no statistically significant cluster is observed during the motor execution state from 0.1 s to 0.6 s.

**Figure 6:**
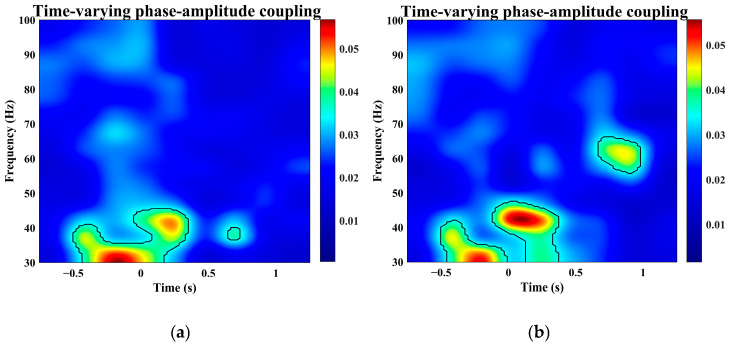
Grand average time-varying θ–γ PAC across all subjects during motor imagery and motor execution tasks. (**a**) θ–γ coupling during the motor imagery task. (**b**) θ–γ coupling during the motor execution task.

Figure 6 illustrates the average time-varying θ–γ PAC across all subjects during the motor imagery and motor execution task conditions. In Figure 6a, which displays the θ–γ coupling during the motor imagery task condition, a highly significant cluster is observed from −0.5 s to 0.3 s (cluster corrected, *p* < 0.01), spanning the high-frequency range of 30–45 Hz. Additionally, a statistically significant cluster is observed from 0.5 s to 0.8 s (cluster corrected, *p* < 0.05), which covers the high-frequency range of 38–42 Hz. By contrast, Figure 6b presents the θ–γ coupling under the motor execution task condition. Highly significant clusters are observed from −0.5 s to 0 s and from −0.1 s to 0.4 s, respectively (cluster corrected, *p* < 0.01), spanning the high-frequency ranges of 30–42 Hz and 30–48 Hz, respectively. Furthermore, a statistically significant cluster is also observed from 0.7 s to 1 s (cluster corrected, *p* < 0.05), covering the high-frequency range of 55–68 Hz.

**Figure 7:**
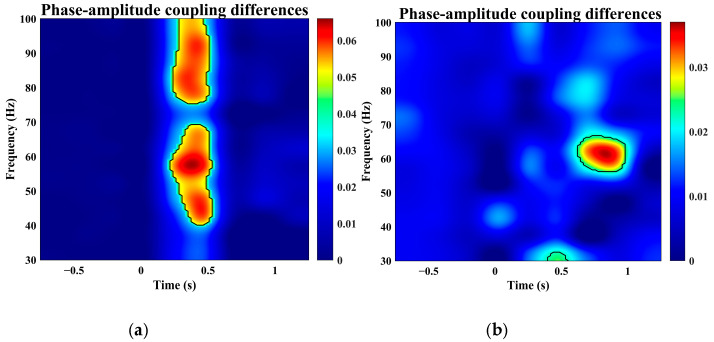
Grand average differences in time-varying PAC between motor imagery and motor execution tasks. (**a**) α–γ coupling differences during the two tasks. (**b**) θ–γ coupling differences during the two tasks.

Figure 7 illustrates the average time-varying PAC differences across all subjects between the motor imagery and motor execution task conditions. In Figure 7a, which displays the α-γ coupling differences between the motor imagery and motor execution task conditions, two highly significant differential clusters are observed from 0.1 s to 0.6 s (corrected *p* < 0.01), spanning the high-frequency ranges of 40–70 Hz and 75–100 Hz, respectively. By contrast, Figure 7b presents the θ–γ coupling differences under the two task conditions. A highly significant differential cluster is observed from 0.6 s to 1.1 s (cluster corrected, *p* < 0.01), which extensively covers the high-frequency range of 57–68 Hz. Furthermore, a statistically significant differential cluster is also observed around 0.5 s (cluster corrected, *p* < 0.05), covering the high-frequency range of 30–32 Hz.

In summary, the statistical analysis of time-varying coupling patterns demonstrates that compared to θ–γ PAC, α–γ PAC appears to provide more information for differentiating between motor imagery and motor execution states. Consequently, we further analyze the comodulogram characteristics of α–γ PAC.

**Figure 8:**
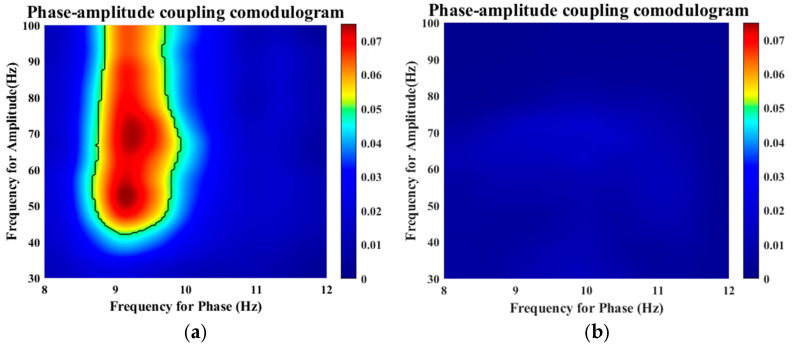
Grand average α–γ PAC comodulograms during motor imagery and motor execution tasks across all subjects. (**a**) α–γ coupling comodulogram during the motor imagery task. (**b**) α–γ coupling comodulogram during the motor execution task.

**Figure 9:**
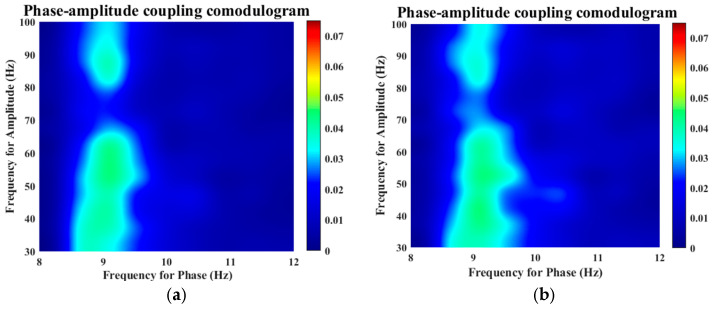
Grand average α–γ coupling comodulograms during the rest period under the two tasks. (**a**) α–γ coupling comodulogram during the rest period of the motor imagery task. (**b**) α–γ coupling comodulogram during the rest period of the motor execution task.

Figure 8 illustrates the grand average α–γ PAC comodulograms during the motor imagery and motor execution tasks across all subjects, where highly significant clusters are observed in the low-frequency range of 8.5–9.5 Hz and high-frequency range of 40–100 Hz (cluster corrected, *p* < 0.01). Figure 9 presents the rest period comodulograms under both the motor imagery and motor execution task conditions across all subjects. Although high-intensity PAC modulation is observed in the ranges of 8.5–9.5 Hz and 30–100 Hz, no statistically significant differential cluster is observed between the rest periods of the motor imagery condition and the motor execution task condition (cluster corrected, *p* > 0.05). This indicates that there is no significant coupling difference between the rest periods of the two conditions.

**Figure 10:**
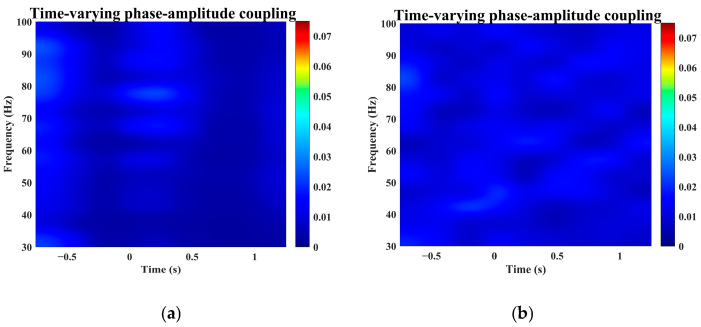
Grand average time-varying α–γ PAC under the motor imagery task condition in two reference regions. (**a**) Coupling effects in the left auditory region. (**b**) Coupling effects in the inferior frontal gyrus.

**Figure 11:**
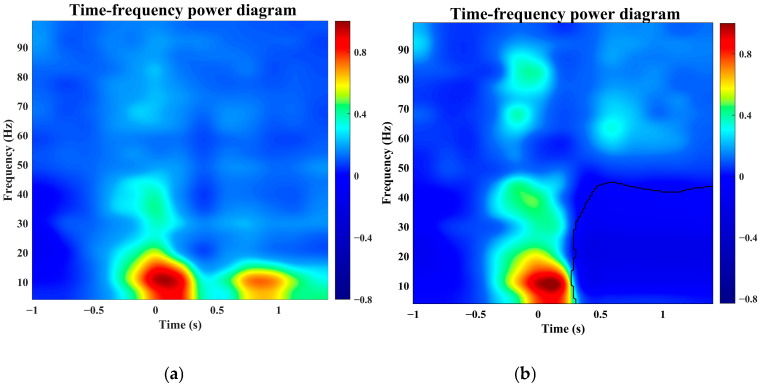
Grand average time-frequency power changes within the regions of interest. (**a**) Power changes under the motor imagery condition. (**b**) Power changes under the motor execution condition.

Figure 10 illustrates the time-varying α–γ PAC effects under the motor imagery task condition in two reference regions, namely the left auditory region and the inferior frontal gyrus. No statistically significant cluster is found in either of these two regions (cluster corrected, *p* > 0.05). This demonstrates that the significant PAC clustering effects and prominent differences observed in the regions of interest are unlikely to be caused by changes in the background magnetic field induced by head movements. Figure 11 displays the grand average time-frequency power changes within the region of interest (ROI) across all subjects under motor imagery and motor execution conditions. These changes were baseline-normalized relative to the mean of the −1 to −0.5 s window prior to cue onset. A highly significant differential cluster is observed from 0.3 s to 1.5 s (cluster corrected, *p* < 0.01), spanning the frequency range of 4–43 Hz. This corresponds to the event-related desynchronization effect under motor conditions described in reference [32]. However, no statistically significant differential cluster is observed during the pre-task time period, which indicates that there is no significant difference in power alterations between the two task conditions prior to the tasks.

### 3.2. Decoding of Motor States Based on PAC Using OPM-MEG

**Figure 12:**
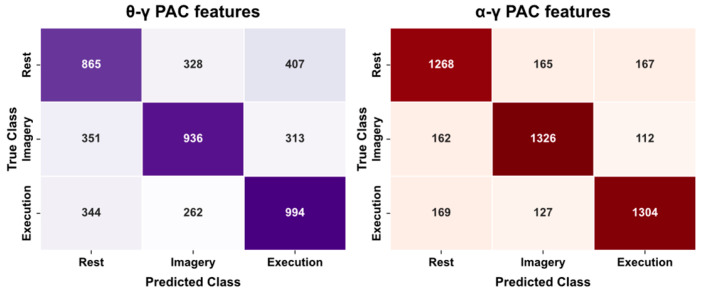
Confusion matrices for motor state decoding based on different PAC features in 10-fold cross-validation.

Figure 12 presents the confusion matrices for motor state classification using θ–γ PAC and α–γ PAC features within a 10-fold cross-validation framework, where the main diagonal indicates correctly classified samples. Evaluated across 1600 samples from 10 subjects, the θ–γ PAC features yielded recall rates of 54.06% for motor imagery, 58.5% for motor execution, and 62.13% for the rest period, culminating in a macro-averaged recall of 58.08% across the three states. Conversely, the α–γ PAC features demonstrated superior performance, achieving recall rates of 79.25% for motor imagery, 82.88% for motor execution, and 81.5% for the rest period, with a macro-averaged recall of 81.21% across the three states. These findings clearly demonstrate that α–γ PAC features possess substantially greater discriminative power for motor state decoding than θ–γ PAC features.

**Figure 13:**
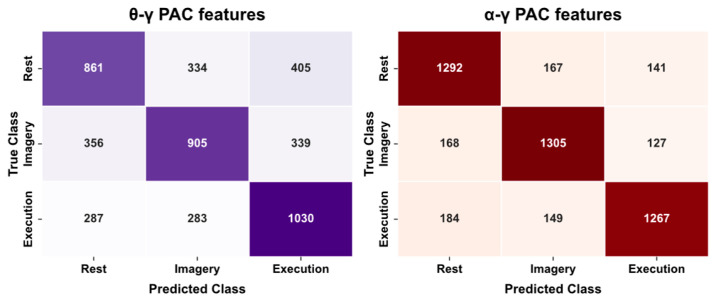
Confusion matrices for motor state decoding based on different PAC features in LOSO cross-validation.

Figure 13 presents the confusion matrices for motor state classification using θ–γ and α–γ PAC features within the LOSO framework. The θ–γ PAC features yielded recall rates of 53.81% for motor imagery, 56.56% for motor execution, and 64.38% for the rest period, culminating in a macro-averaged recall of 58.25% across the three states. Conversely, the α–γ PAC features achieved recall rates of 80.75% for motor imagery, 81.56% for motor execution, and 79.19% for the rest period, with a macro-averaged recall of 80.5% across the three states. Notably, no substantial discrepancy was observed in the macro-averaged recall metrics between the 10-fold and LOSO cross-validation frameworks, indicating that the proposed PAC features possess robust generalization capability across different subjects.

**Figure 14:**
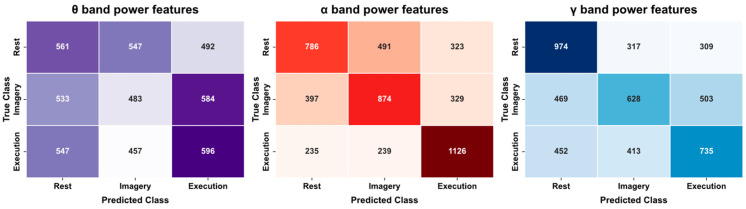
Confusion matrices for motor state decoding based on different power features in 10-fold cross-validation.

**Figure 15:**
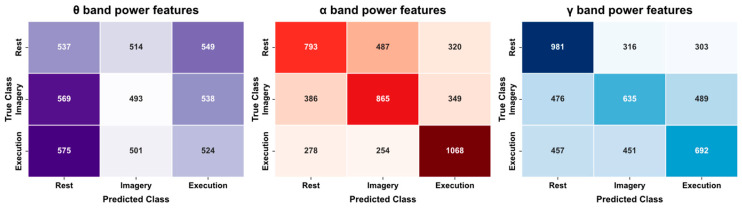
Confusion matrices for motor state decoding based on different power features in LOSO cross-validation.

Figure 14 presents the confusion matrices for motor state classification using θ, α, and γ band power features within a 10-fold cross-validation framework. The macro-averaged recall across the three states reached 34.17% for θ band power, 58.04% for α band power, and 48.69% for γ band power features. For comparison, Figure 15 illustrates the classification confusion matrices under the LOSO cross-validation framework. Under this setup, the θ, α, and γ band power features yielded macro-averaged recalls of 32.38%, 56.79%, and 48.08%, respectively. Similarly, no substantial discrepancy was observed in the macro-averaged recall metrics between the 10-fold and LOSO cross-validation frameworks. This outcome indicates that the proposed band power features possess robust generalization capability across different subjects.

In summary, within the scope of this study, despite the fact that the α and γ band power features possess state-decoding capabilities with macro-averaged recalls above the 33% chance level, they are outperformed by the PAC features. Specifically, within the 10-fold cross-validation framework, the θ–γ PAC features yielded an absolute increase of 23.91% and 9.39% over the θ and γ band power features, respectively, while the α– γ PAC features showed an absolute increase of 23.17% and 32.52% over the α and γ band power features. Similarly, under the LOSO cross-validation setup, the θ–γ PAC features exhibited an absolute increase of 25.87% and 10.17% compared to the θ and γ band power features, respectively, whereas the α–γ PAC features achieved an absolute increase of 23.71% and 32.42% over the α and γ band power features. Importantly, beyond the macro-averaged recall, other macro-averaged metrics calculated from the confusion matrices also demonstrated superior performance compared to single-band power features. This indicates that cross-frequency band interactions potentially harbor richer information regarding motor states.

**Figure 16:**
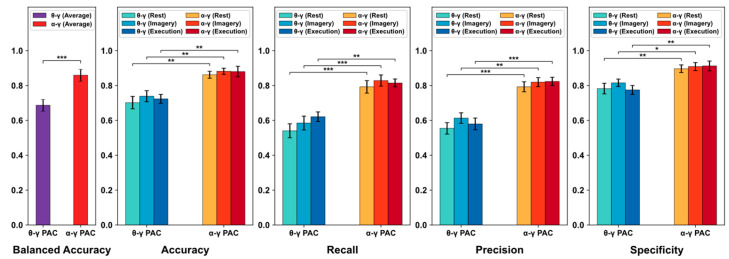
Comparison of decoding performance using different PAC features in 10-fold cross-validation. *, **, and *** indicate statistical significance at *p* < 0.05, *p* < 0.01, and *p* < 0.001, respectively.

Figure 16 presents the statistical comparison of decoding performance between θ–γ PAC and α–γ PAC features across 10-fold cross-validation. The group-level balanced accuracy was computed as the mean across all motor states, yielding 68.67% ± 1.32% for θ–γ PAC and 85.91% ± 1.14% for α–γ PAC. Wilcoxon signed-rank tests with Bonferroni correction revealed that α–γ PAC significantly outperformed θ–γ PAC across all evaluated metrics. Specifically, for balanced accuracy (averaged across all states: *p* < 0.001), accuracy (all states: *=*<0.01), recall (Resting and Motor Imagery: *p* < 0.001; Motor Execution: *p* < 0.01), precision (Resting and Motor Execution: *p* < 0.001; Motor Imagery: *p* < 0.01), and specificity (Resting and Motor Execution: *p* < 0.01; Motor Imagery: *p* < 0.05).

**Figure 17:**
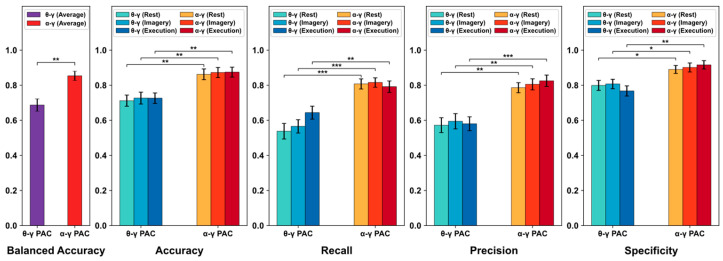
Comparison of decoding performance using different PAC features in LOSO. *, **, and *** indicate statistical significance at *p* < 0.05, *p* < 0.01, and *p* < 0.001, respectively.

Figure 17 presents the statistical comparison of decoding performance between θ–γ PAC and α–γ PAC features under leave-one-subject-out (LOSO) cross-validation. The group-level average balanced accuracy was 68.69% ± 1.25% for θ–γ PAC and 85.38% ± 1.07% for α–γ PAC. Wilcoxon signed-rank tests with Bonferroni correction again revealed that α–γ PAC significantly outperformed θ–γ PAC across all performance metrics, including balanced accuracy (mean across all states: *p* < 0.01), accuracy (all states: *p* < 0.01), recall (Resting and Motor Imagery: *p* < 0.001; Motor Execution: *p* < 0.01), precision (Resting and Motor Imagery: *p* < 0.01; Motor Execution: *p* < 0.001), and specificity (Resting and Motor Imagery: *p* < 0.05; Motor Execution: *p* < 0.01).

## 4. Discussion

Previous studies have demonstrated that phase–amplitude coupling (PAC) plays a crucial role in neuronal encoding and information processing. It has been widely used to reveal sequence information encoding [33], functional interactions across brain regions [34], and sensory information integration processes [35], and has gradually emerged as a potential neurophysiological biomarker in studies of disorders such as Alzheimer’s disease [36], autism spectrum disorder [37], schizophrenia [38], and Parkinson’s disease [39]. However, studies on PAC features measured using OPM-MEG for motor-state decoding are still lacking. Therefore, in this study, we evaluated the decoding performance of two PAC features derived from OPM-MEG across three conditions: rest period, motor imagery, and motor execution. The results demonstrate that PAC features within specific coupling frequency bands measured by OPM-MEG can be effectively applied to motor state decoding, with the α–γ PAC feature achieving the best performance in this study.

Studies by Gwon et al. [40] have shown that the α rhythm is closely associated with motor imagery, execution, and observation. In fact, α power decreases before and after motor events and then rebounds to baseline levels. Therefore, the α rhythm is central to understanding motor function and has become a key feature in the construction of brain–computer interfaces, such as those based on motor imagery. Research by Nowak et al. [41] demonstrated that γ-band oscillatory activity is enhanced during human motor processes, with increases in γ amplitude observed in both cortical and subcortical regions during motor execution, suggesting a critical role for γ oscillations in movement. Du et al. [13] reported that, during the preparatory period of motor tasks, γ amplitude on movement-selective electrodes exhibits strong coupling with the α phase, maintaining high γ amplitude in a phase-dependent manner. This coupling gradually weakens as movement is executed, and high γ amplitude becomes less phase-dependent. Collectively, these previous studies indicate that α and γ rhythms play crucial roles in motor imagery and execution, and that the coupling between γ amplitude and α phase may provide key information about motor states. These findings partially support the decoding performance of α–γ PAC features derived from OPM-MEG signals observed in the present study.

Studies by Horschig [42] and Brauns [43] et al. have shown that θ and α activity play important roles in motor planning and execution, supporting the integration of perceptual and motor information necessary for movement. Marzulli et al. [44] demonstrated that θ–γ coupling, in which the phase of θ oscillations modulates the amplitude of γ oscillations, serves as a neural coding mechanism reflecting coordinated information processing across brain regions. They observed transient, time-locked changes in θ–γ coupling during the planning and imagination of multiple sequential actions. Previous studies have also indicated that θ–γ coupling is involved in memory processes and sensorimotor tasks. Together, these findings suggest that θ–γ coupling contributes to motor-state recognition, consistent with the θ–γ coupling decoding results observed in the present study.

The biophysical mechanism underlying the observed PAC within the motor cortex can be explained by a cross-frequency gating by inhibition framework [14,45]. From a physiological perspective, the phase of low-frequency rhythms reflects macroscopic, periodic fluctuations in cortical excitability driven by the thalamocortical loop, where the specific phase indicates a temporary window of open excitability [46,47]. Conversely, the amplitude of the γ band physically indexes the dense localized population spiking and fast synchronization within cortical microcircuits [48,49]. Within this framework, the macro-scale excitability cycle of the low-frequency phase rhythmically modulates and gates the timing of localized computational packages in the γ amplitude, thereby achieving precise coordination of local motor-related processing [27,39,50]. This cross-frequency coordination manifests as a prominent coupling during motor planning to suppress premature outputs, followed by a dynamic attenuation and reconfiguration during execution to facilitate the transition between different motor states [12,13,51].

The stronger alpha-gamma PAC during motor imagery appears counterintuitive given that motor execution involves greater overt cortical activation. However, this asymmetry aligns with the hold-and-release mechanism proposed by Yamashita et al. [14]: α–γ PAC in the sensorimotor cortex serves to hold high γ motor representations in a phase-dependent state during preparation, and releases them as alpha power desynchronizes during execution. Motor imagery constitutes an internal simulation that functionally resembles the motor preparation period [52], where α–γ coupling remains sustained to maintain the motor plan under inhibitory control without overt execution. Gwon et al. [40] reported that α–γ PAC decreases during motor imagery in correlation with α ERD, suggesting that the pre-imagery coupling strength determines the subsequent release dynamics. This is consistent with the hold and release framework: stronger initial coupling during the ready period leads to a more pronounced decrease during imagery, reflecting the transition from holding to releasing the motor plan. Conversely, during actual execution, alpha ERD weakens the phase modulation, shifting the system toward phase-independent high γ encoding. Thus, the stronger α-γ PAC during imagery reflects a prolonged holding state characteristic of internal simulation, not stronger motor cortical activation.

We acknowledge a limitation in the characterization of the rest period in this study, where the feature window was defined as the −500 to 0 ms interval relative to cue onset. Given the regular trial structure, participants might have entered a state of preparatory attention during this pre-cue interval, raising concerns about potential contamination from anticipatory neural activity or carry-over effects. Nevertheless, the following observations help alleviate these concerns: (a) no warning signal was presented before the auditory cue, thereby minimizing explicit motor preparation; (b) the observed time-varying PAC patterns during the rest period were highly similar between the two distinct task conditions (see Figure 6 and Figure 7), with cluster-based permutation tests revealing no significant differences in their temporal profiles; (c) cluster-based permutation tests of the baseline comodulograms showed no significant clusters differentiating the rest-period PAC between the motor imagery and motor execution conditions (see Figure 9); and (d) time-frequency analysis using cluster-based permutation tests revealed no significant clusters of power differences between the two conditions during the rest period, demonstrating very similar baseline power patterns (see Figure 11). Together, these findings indicate that our baseline data were not significantly contaminated by task-specific anticipatory attention or motor preparation.

Additionally, although a priori power analysis suggested that the current sample size was acceptable for the present research objectives, we acknowledge that recruiting a larger cohort in future studies would be beneficial to further strengthen the generalizability of the findings. The participant sample also exhibited a skewed sex distribution (male: female = 4:1), which may limit the extrapolation of the results to female populations. In future work, we will endeavor to optimize recruitment strategies, expanding the sample size while striving for a more balanced sex representation, to mitigate these limitations.

## 5. Conclusions

In this study, the OPM-MEG system was employed to record brain responses during motor imagery and motor execution tasks. The feasibility and accuracy of using PAC features derived from OPM-MEG for motor state decoding were systematically evaluated. The results demonstrated that PAC features within specific frequency bands could successfully discriminate among rest period, motor imagery, and motor execution, with α–γ PAC exhibiting the highest performance. These findings provide evidence that OPM-MEG can reliably capture motor task-related PAC features and highlight its potential for state decoding applications. Looking ahead, this technology could be further extended to research on brain–computer interface-based motor control, as well as studies of movement disorders such as Parkinson’s disease and multiple sclerosis, thereby offering broader opportunities for investigating PAC mechanisms using OPM-MEG.

## Data Availability

The datasets are not publicly available due to privacy and ethical restrictions. However, partial datasets may be available upon reasonable request. Requests for access to the datasets should be directed to Y. Li (email: by2017335@buaa.edu.cn).

## Appendix A

**Table A1:** Benchmarking of balanced accuracy performance for motor state decoding across different classifiers.

Feature Type	Cross-Validation Method	LDA	SVM	RF
θ–γ PAC	10-fold	68.67%	68.30%	68.36%
	LOSO	68.69%	68.81%	68.63%
α–γ PAC	10-fold	85.91%	85.77%	85.52%
	LOSO	85.38%	85.52%	85.16%
θ band power	10-fold	50.63%	50.55%	50.39%
	LOSO	49.28%	49.34%	49.41%
α band power	10-fold	68.53%	68.53%	68.33%
	LOSO	67.29%	67.45%	67.23%
γ band power	10-fold	61.52%	61.44%	61.31%
	LOSO	61.06%	61.09%	60.97%
